# The Systemic Immune Network in Recent Onset Type 1 Diabetes: Central Role of Interleukin-1 Receptor Antagonist (DIATOR Trial)

**DOI:** 10.1371/journal.pone.0072440

**Published:** 2013-08-26

**Authors:** Hubert Kolb, Kathrin Lückemeyer, Tim Heise, Christian Herder, Nanette C. Schloot, Wolfgang Koenig, Lutz Heinemann, Stephan Martin, on behalf of the DIATOR Study Group

**Affiliations:** 1 West-German Centre of Diabetes and Health, Verbund Katholischer Kliniken Düsseldorf, Düsseldorf, Germany; 2 Profil Institute for Metabolic Research, Neuss, Germany; 3 Institute for Clinical Diabetology, German Diabetes Center, Leibniz Center for Diabetes Research at Heinrich Heine University, Düsseldorf, Germany; 4 Department of Internal Medicine II - Cardiology, University of Ulm Medical Center, Ulm, Germany; La Jolla Institute for Allergy and Immunology, United States of America

## Abstract

**Background:**

The hypothesis was tested that the systemic immune milieu in recent-onset type 1 diabetes is associated with residual beta cell function and other metabolic patient characteristics.

**Methods and Findings:**

All patients (n = 89, 40% female) of the Diabetes and Atorvastatin (DIATOR) Trial were analyzed at recruitment, i.e. prior to receiving the study medication. Inclusion criteria were insulin dependent diabetes for 2 weeks to 3 months, age range 18–39 years, and islet cell autoantibodies. Blood samples were analyzed for 14 immune mediators by standard methods. Concentrations of all mediators correlated with at least one other mediator (p<0.05, Spearman correlation) giving rise to a network. Interleukin 1 receptor antagonist (IL1-RA) held a central position and was associated with both pro- and anti-inflammatory mediators. Further central elements were the pro-inflammatory mediators CRP and IL-6, the soluble adhesion molecules sICAM-1 and E-selectin, and MCP-4 which held a central position in the chemokine network. The two Th1-associated mediators IFNγ and IP-10 remained outside the network but correlated with each other. All correlations were positive (r = 0.25–0.72), i.e., high levels of pro-inflammatory mediators were accompanied by increased levels of anti-inflammatory mediators. IL-1RA was the only mediator associated with fasting and liquid mixed meal stimulated C-peptide concentrations (r = 0.31 and 0.24, p = 0.003 and 0.025, after adjustment for age, sex, BMI). There were associations between the immune mediator network and BMI (IL-1RA, CRP, IL-6, MCP-4, MIP-1ß) but few or no associations with HbA1c, insulin dose, lipid parameters, age or sex.

**Conclusions:**

In patients with recent onset type 1 diabetes, systemic acute phase proteins, cytokines, chemokines and soluble adhesion molecules form a network. Among the few central elements IL-1RA has a dominant role. IL-1RA is associated with all other groups of mediators and is the only mediator which correlates (positively) with residual beta cell function.

**Trial registration:**

ClinicalTrials.gov registration number: NCT00974740

## Introduction

Type 1 diabetes is an immune-mediated disease of which the exact pathomechanism remains to be elucidated [1]–[3]. Since the target tissue is small and sequestered within the pancreas gland there may not be a strong relationship between local immune activity and the systemic immune milieu. However, at the level of circulating immune mediators previous studies have indicated a relationship between serum concentrations of interleukin 1 receptor antagonist (IL-1RA) and residual beta cell function in the first year after diagnosis with type 1 diabetes [4]. These studies were limited by the low number of immune mediators analyzed. We therefore made use of the data set generated by an intervention trial with atorvastatin in patients with recent-onset type 1 diabetes to study in detail the association of immune mediators with beta cell function and other metabolic and general patient characteristics.

The immune mediators analyzed included the acute phase response, pro- and anti-inflammatory activity as well as Th1 and Th2 immunity. Limitations were the availability of assays requiring only small serum quantities or the lack of detectable signals in a major fraction of patients. C-reactive protein (CRP) is a lead component of the acute phase response [5]. Immune mediators with preferential pro-inflammatory activity are interleukin (IL)-6 [6], IL-18 [7], IL-8 (chemokine CXCL8) [8], macrophage chemoattractant protein (MCP)-1 (CCL2) [9], macrophage inflammatory protein (MIP)-1ß (CCL4) [10]. Human MCP-4 (CCL13) appears to promote both, Th1 and allergic inflammation [11], [12]. Macrophage-derived chemokine (MDC, CCL22) and thymus and activation regulated chemokine (TARC, CCL17) mediate Th2 dependent immune responses and inflammation [13], [14], whereas interferon (IFN) gamma inducible protein (IP)-10 (CXCL10) and IFNγ are central mediators of Th1 dependent immunity [15], [16].

The soluble adhesion molecules soluble intercellular adhesion molecule (sICAM)-1 and endothelial selectin (E-selectin)-1 are biomarkers of endothelial dysfunction [17]–[19]. They are thought to promote inflammation, but sICAM-1 has also been shown to interfere with binding of leukocytes to the endothelium and with the development of autoimmune diabetes in animal models [20]–[22]. IL-1RA is a potent anti-inflammatory and regulatory immune mediator [23].

In addition to analyzing the relationship between these immune mediators and patient characteristics we searched for associations between the 14 mediators to learn about possible regulatory mechanisms.

## Results

### Characteristics of the Systemic Immune Mediator Network

In order to analyze for associations between systemic immune mediators correlations were calculated for all possible pairs of the 14 mediators measured. Since serum concentrations of some mediators were not normal or lognormal distributed, and for minimizing the effect of outliers, the Spearman rank correlation test was applied. No α-adjustment was performed.

**Figure 1:**
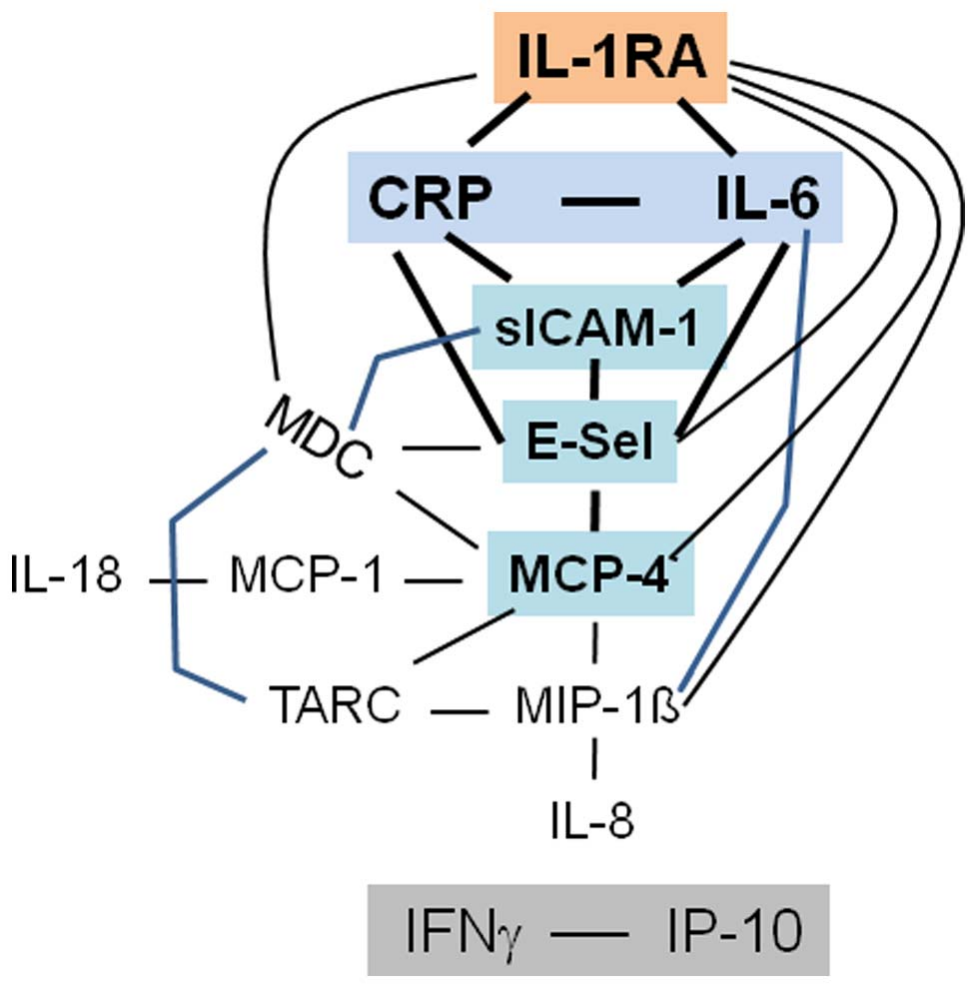
Systemic immune mediator network in patients with recent onset type 1 diabetes. Shown are all significant correlations (p<0.05) between single immune mediators, Spearman rank correlation test. Lines crossing other lines are indicated in blue. Stronger lines indicate associations between central elements of the network. Central elements and the second network are shaded for better readability. Correlation coefficients are listed in Table 1.

Only positive associations were found, even between pro- and anti-inflammatory mediators such as IL-6 or CRP and IL-1RA (**Table 1**). The graphic representations of all significant correlations yielded a scheme with IL-1RA as central mediator which was directly linked with many pro- and anti-inflammatory mediators (**Fig. 1**). Two additional core elements of the network were CRP+IL-6 as well as sICAM-1+ E-selectin (Fig. 1). MCP-4 was a central element of the chemokine part of the network. The two Th1 immunity-associated immune mediators IFNγ and IP-10 remained outside the network but were associated with each other. Correlation coefficients ranged between 0.25 and 0.72.

**Table 1:** Correlation analysis of immune mediator blood levels^a^.

	IL-1RA	CRP	IL-6	sICAM-1	E-Sel	MCP-4	MDC	MIP-1ß	MCP-1	IL-18	TARC	IL-8	IFNγ	IP-10
**IL-1RA**	1	0.38d	0.45		0.25	0.33b	0.35	0.29b						
**CRP**		1	0.45	0.36	0.51									
**IL-6**			1	0.25	0.31b,d			0.32						
**sICAM-1**				1	0.47		0.27							
**E-Sel**					1	0.43	0.31							
**MCP-4**						1	0.40	0.28^b^	0.36		0.72			
**MDC**							1				0.48			
**MIP-1ß**								1			0.31	0.27		
**MCP-1**									1	0.26				
**IL-18**										1				
**TARC**											1			
**IL-8**												1		
**IFNγ**													1	0.33
**IP-10**														1

a Shown are correlation coefficients (r) for pairs of immune mediators with significant correlation p<0.05, Spearman rank correlation.

b p-value >0.05 after adjustment for BMI.

c p-value >0.05 after adjustment for age.

d p-value >0.05 after adjustment for sex.

Of the 25 significant associations depicted in **Fig. 1** only one was lost after adjustment for age, and four were lost after adjustment for BMI (**Table 1**). The network was not different between males and females, i.e., adjustment for sex affected only two associations.

### Association of the Systemic Immune Mediator Network with Beta Cell Function

The central role of IL-1RA in the immune mediator network was underscored by the finding of an association with fasting and stimulated serum C-peptide concentrations (**Table 2**). The association with C-peptide concentrations before and after a standard liquid mixed meal was attenuated by adjusting for age, sex and BMI but remained significant. Systemic concentrations of CRP also correlated with fasting and stimulated C-peptide levels, but this association lost significance after adjustment. The 12 other immune mediators measured did not correlate with fasting or stimulated serum C-peptide levels (**Table 2**).

**Table 2:** Association of immune mediator blood levels with residual beta cell function.

Immunemediator	Correlation^a^ with			
	fasting C-peptide		stimulated C-peptide^b^	
	r	p-value	r	p-value
**IL-1 RA**	0.31	0.003	0.24	0.025
p adjusted^c^		0.027		0.044
**CRP**	0.28	0.011	0.22	0.044
p adjusted^c^		>0.05		>0.05

a Spearman rank correlation.

b 90 min after standard liquid mixed meal.

c adjusted for age, sex, BMI.

All other immune mediators were not correlated, p>0.05.

In order to test further for an association of immune mediators with beta cell function we performed two types of analyses. In a first analysis we compared serum C-peptide levels between patients who generally had high systemic concentrations of immune mediators with those exhibiting lower concentrations. For each of the 14 immune mediators we categorized a patient as exhibiting a serum concentration above or at/below the median. Patients with generally low immune mediator levels had above median concentrations for only very few immune mediators, patients with generally high immune mediator levels exhibited above median concentrations for most immune mediators. The number of immune mediators above median did not correlate with the corresponding fasting or stimulated C-peptide concentrations (both r = 0.19, p = 0.09, Spearman rank correlation test). I.e., concentrations of fasting or meal-stimulated serum C-peptide were not significantly different between patients with preferentially high versus low immune mediator levels.

In a second analysis we searched for an association between C-peptide concentrations and combinations of selected immune mediators. In none of the cases did the addition of a second or of further immune mediators to IL-1RA strengthen the association with fasting or stimulated C-peptide levels (data not shown).

### Association of the Systemic Immune Mediator Network with Metabolic or General Characteristics

There were only few associations of systemic immune mediator concentrations with metabolic or general characteristics, except for BMI. Among patients BMI positively correlated with blood concentrations of IL-1RA, CRP and IL-6, and this was also seen for the two chemokines MCP-4 and MIP-1ß (**Table 3**), also after adjustment for age and sex of patients. HbA1c values correlated with sICAM-1 and MCP-1 only, and insulin dose with none of the 14 immune mediators (**Table 3**). Blood lipid levels showed little association with the immune mediator network, total or LDL-cholesterol levels did not correlate with immune mediator levels, HDL-cholesterol concentrations were found negatively associated with IL-6. Triglyceride levels did not exhibit associations with immune mediators (**Table 3**).

**Table 3:** Association of immune mediator blood levels with metabolic characteristics or age.

Characteristic	Immune mediator	Correlation^a^	
		r	p-value
**BMI**	IL-1RA	0.40	0.001
	CRP	0.40	0.001
	IL-6	0.30	0.017
	MCP-4	0.33	0.01
	MIP-1ß	0.28	0.029
	All other		>0.05
**HbA1c**	sICAM-1	0.28	0.038
	MCP-1	0.33	0.014
**HDL-cholesterol**	All		>0.05
**Insulin dose** b	All		>0.05
**Total cholesterol**	All		>0.05
**LDL-cholesterol**	All		>0.05
**HDL-cholesterol**	IL-6	−0.37	0.003
	All other		>0.05
**Triglycerides**	All		>0.05
**Age** c	MDC	−0.30	0.018
	All other	0.30	>0.05

a Spearman rank correlation, adjusted for age, sex and BMI, as appropriate.

b mean insulin dose per day.

c mean age of patients 29.9±6.7 years (SD).

**Figure 2:**
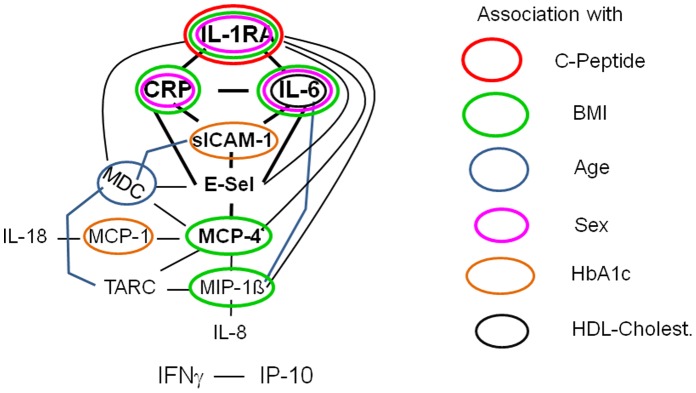
Association of the immune mediator network with metabolic and general characteristics. Associations were found primarily with core elements of the network, with a major role of IL-1RA. Peripheral elements of the network show only few correlations of systemic concentrations with metabolic parameters, and none with general characteristics. Blood lipid parameters show no association with immune mediators, with one exception. All correlations (Spearman rank) with p<0.05 after adjustment for age, sex and BMI, as appropriate, are shown. Central elements and the second network are shaded for better readability.

Mean concentrations of IL-1RA, CRP and IL-6 were higher in females, and the differences remained significant after adjustments for age and BMI. No significant associations were seen with the age of patients except for a negative association with concentrations of MDC (**Table 4**). Graphic representation of the associations found showed that multiple associations were only observed for core element of the immune mediator network, IL-1RA, CRP and IL-6 (**Fig. 2**).

**Table 4:** Comparison of immune mediator blood levels between female and male patients.

Immune mediator	Female	Male	p-Value
**IL-1RA** (pg/ml)^a^	459	294	0.0004
**CRP** (mg/ml)^a^	1.98	1.08	0.004
**IL-6** (pg/ml)^a^	1.61	0.79	0.024
**All other**			>0.05

a mean value adjusted for age and BMI.

## Discussion

Correlations between the systemic concentrations of 14 immune mediators in patients with recent onset type 1 diabetes yielded a web with central and peripheral elements. A central position was found for IL-1RA which was the only mediator associated with the pro-inflammatory core elements CRP+IL-6, with soluble adhesion molecules (E-selectin), and with several chemokines (MCP-4, MIP-1ß, MDC). The Th1 immunity-associated cytokine IFNγ and chemokine IP-10 did not associate with the network but remained separate.

In view of the many immune parameters analyzed, significant correlation coefficients may have occurred by chance in this descriptive analysis. A biological relevance of most r-values is supported by the observation of significant correlations of individual blood levels between pairs of immune mediators known to share regulatory control. All chemokines (MCP-4, MIP-1ß, IL-8, MDC, MCP-1, TARC) were found to be associated with the core chemokine MCP-4 or with MIP-1ß. The exception is IP-10, which is known to be induced by Th1 cytokine IFNγ [24], [25]. Indeed, systemic concentrations of IP-10 and IFNγ were correlated. The Th2 type chemokines MDC and TARC have been described to be regulated in parallel [14], and their concentrations correlated in the current analysis. IL-6 has been reported to regulate the expression of CRP [26], and this fits with the association of systemic concentrations of the two immune mediators observed here. Soluble adhesion molecules are often expressed in parallel [27], [28], and this is reflected by the association between blood levels of sICAM-1 and E-selectin in our analysis.

In cases of moderate upregulation of circulating levels of CRP and IL-6, such as in subclinical systemic inflammation of type 2 diabetes, this is usually accompanied by increased concentrations of soluble adhesion molecules [29]. The association found here for both CRP and IL-6 with the two soluble adhesion molecules sICAM-1 and E-selectin probably indicates similar co-regulation of these four immune mediators. However, the associations observed do not seem to be mediated by the obesity status of patients. After statistical adjustment for individual BMI five of the six associations between CRP, IL-6, sICAM-1 and E-selectin remained significant, only the association between IL-6 and E-selectin was lost. For many significant associations between immune mediators the observed correlation coefficients were below 0.5. This was also the case for pairs of immune mediators which are known to be co-regulated, such as IFNγ and IP-10 (r = 0.33) or CRP and IL-6 (r = 0.45). In persons with type 2 diabetes or impaired glucose tolerance, the correlation coefficient for CRP and IL-6 was reported to be in a similar range (r = 0.49) [30]. Additional internal and external factors appear to contribute to the regulation of systemic concentrations of immune mediators.

A remarkable finding is that all correlations found are positive, also between putative antagonistic pro-inflammatory and anti-inflammatory mediators (such as CRP versus IL-1RA). This is the characteristic of a highly buffered system where increased production of agonistic mediators is counterbalanced by increased levels of antagonistic mediators. Because of its central position, levels of IL-1RA may be particularly relevant for limiting aggressive/inflammatory immune reactivity. This is supported by the observation that parameters of beta cell function (fasting and meal stimulated serum C-peptide concentrations) associate only with IL-1RA and with none of the other 13 immune mediators analyzed, after adjustment for age, sex and BMI. The positive association of IL-1RA blood levels with beta cell function has been observed also in a cohort of children with type 1 diabetes, during the first year after diagnosis [4]. In the latter study, a negative association of stimulated C-peptide concentrations was found during the first year after diagnosis with the pro-inflammatory Th1-associated chemokine MIP-1α (CCL3) [31]. Since MIP-1α was not included in the panel of immune mediators measured in the context of the DIATOR study, its position in the immune mediator network remains to be determined.

The clinical relevance of the association of serum IL-1RA concentrations with beta cell function is uncertain. In rheumatoid arthritis the administration of recombinant IL-1RA is clinically effective in dampening local inflammation although clinical efficacy is less than seen for TNF blocking agents [32]. Down regulation of systemic inflammation by treatment with IL-1RA was also observed in patients with long standing type 2 diabetes, and there was significant preservation of residual beta cell function, lasting for at least 9 months after the end of treatment, in the majority of patients [33], [34]. However, administration of the antagonist protein in patients with recent onset type 1 diabetes did not result in improved beta cell function or other clinical parameters, although the treatment protocol resembled that being effective in rheumatoid arthritis [35].

It is noteworthy that residual beta cell function in patients with recent onset type 1 diabetes is associated with systemic concentrations of an inhibitor of pro-inflammatory or aggressive immune reactivity. IL-1RA suppresses central pathways of inflammatory and destructive immunity via blockade of the IL-1 receptor [36]. Hence, the extent of beta cell destruction may be determined by the ability of the organism to contain aggressive immunity. The ability to respond to beta cell destructive processes by up-regulating counter-reactive protective mechanisms may also be the reason behind resistance to type 2 diabetes in healthy obese people [37].

The association between beta cell function and circulating immune mediators appears to change with the progression of type 1 diabetes. There is substantial loss of beta cell function and probably of beta cell mass in the years following diagnosis [38], and a regression of the insulitis process [39]. Islet inflammation was found to persists when there was still substantial beta cell mass present [38]. Indeed, we found in longer term patients with type 1 diabetes a positive association of residual beta cell function with pro-inflammatory immune mediators IL-6 and TNFα, whereas increased concentrations of anti-inflammatory/regulatory immune mediators (Il-1RA, IL-10, transforming growth factor-ß1 and -ß2) were seen in patients with low serum C-peptide concentrations and assumed little residual beta cell mass [40].

We also searched for associations between the systemic immune mediator network and further metabolic or general patient characteristics. There were only a small number of significant associations except for BMI. Similar as reported from studies in obese persons, BMI correlated with CRP, IL-6, IL-1RA, MIP-1ß, MCP-4 [41]–[46]. HbA1c levels correlated with systemic concentrations of the two soluble adhesion molecules. Since both mediators are markers of endothelial dysfunction, increased concentrations may be expected with poor metabolic control [47], [48]. No association was found for insulin dose. Blood lipid parameters also showed no association with immune mediators, with one exception, IL-6. The negative association of IL-6 with HDL-cholesterol fits with the positive association of IL-6 with BMI [41], [42]. An association with sex was observed for IL-1RA, CRP and IL-6, with higher immune mediator levels in female patients. In a large case-chort and a population-based study similar results were obtained for IL-1 RA and IL-6, respectively, but not for CRP [46], [42]. Of the 14 immune mediators only MDC concentrations were (negatively) correlated with age. The limited age range of 18–39 years for patient recruitment probably did not allow to detect the increase of blood levels of pro-inflammatory immune mediators seen with advanced age [49]. Graphic presentation of these findings (Fig. 2) shows that peripheral elements of the network exhibit only few correlations with metabolic parameters, and none with general characteristics. Multiple associations are seen only for core elements of the network, IL-1RA, CRP and IL-6.

In conclusion, acute phase proteins, cytokines, chemokines and soluble adhesion molecules show significant correlations of their blood levels in patients with recent-onset type 1 diabetes. Among the core elements of the network IL-1RA has a dominant role, being associated with all groups of immune mediators. The network appears to be regulated, i.e., high levels of pro-inflammatory mediators are accompanied by high levels of antagonistic mediators. The central role of IL-1RA is supported by the finding that it is the only mediator showing association with residual beta cell function. There are only few significant correlations between concentration of immune mediators and other metabolic or general patient characteristics.

## Methods

### Trial Procedure and Laboratory Analyses

Patients with newly diagnosed type 1 diabetes were recruited in 12 German centers. Inclusion criteria were insulin dependent diabetes for two weeks to 3 months, age range 18–39 years, and at least one islet autoantibody (to glutamic acid decarboxylase 65 or to insulinoma-associated antigen 2, or islet cell antibodies). Patients were recruited for the DIATOR Trial which investigated the possible impact of treatment with atorvastatin on beta cell function [50]. In the current analysis, only the blood samples obtained prior to treatment with atorvastatin or placebo were considered.

The study was conducted in accordance with the Declaration of Helsinki, and approval by the ethics committee of the Ärztekammer Nordrhein was obtained. All patients provided informed written consent prior to study entry. All parts of the study were conducted in Germany. The study protocol and the informed written consent included measurement and analyses of immune mediators as reported here. The study protocol is available as supporting information S1 to ref. 50.

Plasma C-reactive protein (CRP) concentrations were determined by an immunonephelometric assay [51]. Serum concentrations of sICAM-1, E-selectin, IL-6, IL-1RA, IFNγ, IP-10, MCP-4, MIP-1ß, MDC and TARC were measured by double-antibody ELISA, IL-18, MCP-1 and IL-8 were determined by bead-based multiplex technology [50], [52].

Stimulated C-peptide secretion was assessed using serum C-peptide concentrations after a standardized liquid mixed meal (Boost HP® (Mead Johnson, Evansville, IN, USA), 6 ml per kg body weight with a maximum of 360 ml) [53]. C-peptide was measured in serum by an immunoenzymatic assay (Biosource/Invitrogen, Karlsruhe, Germany). Biochemical blood parameters were analyzed in a central laboratory.

### Statistical Analysis

Data were analyzed using SAS® System for Windows (Version 9.3). Analyses were performed in all subjects at baseline. For correlation analyses, as not all parameters were normally distributed, Spearman rank correlation test was performed for all parameters. To examine stability of the correlated parameters, these correlations were adjusted by age, BMI and sex – as appropriate. For regression analyses a forward selection was used to find the best associated immune mediators to beta cell function. An ANOVA was used to compare the effects of gender on the different immune mediators. The level of significance was set at 0.05.
